# The 3-dimensional miniplate is more effective than the standard miniplate for the management of mandibular fractures: a meta-analysis

**DOI:** 10.1186/s40001-017-0244-2

**Published:** 2017-02-14

**Authors:** Yong Liu, Bo Wei, Yuxiang Li, Dawei Gu, Guochao Yin, Bo Wang, Dehui Xu, Xuebing Zhang, Daliang Kong

**Affiliations:** 1 Departments of Orthopaedics, Jilin Oilfield General Hospital, Songyuan, 131200 China; 2 Departments of Neurosurgery, China-Japan Union Hospital of Jilin University, Changchun, 130033 Jilin China; 3 Departments of Orthopaedics, China-Japan Union Hospital of Jilin University, Changchun, 130033 Jilin China

**Keywords:** Mandibular fractures, Standard miniplate, 3-Dimensional miniplate, Complication rates, Meta-analysis, Subgroup analysis

## Abstract

**Purpose:**

The study aimed to determine the superiority between 3-dimensional (3D) miniplate and standard miniplate for mandibular fractures (MFs) treatment.

**Background:**

Controversial results on the use of standard miniplate and 3D miniplate have remained for management of MFs.

**Methods:**

Several electronic databases were retrieved up to September 2014 to identify eligible studies. The quality of studies was assessed, and the relative risk (RR) with its corresponding 95% confidence interval (CI) was assessed to measure the effect size. Subgroup analyses by different fracture regions and different 3D miniplate sizes were performed. Publication bias was measured by a funnel plot.

**Results:**

There were 13 studies included for the meta-analysis, consisting of 593 participants. The 3D miniplate achieved significant lower incidences of malocclusion (RR 0.43, 95% CI 0.24–0.77, *P* = 0.004) and hardware failure (RR 0.31, 95% CI 0.13–0.74, *P* = 0.008) than the standard miniplate. There were no significant differences between the two miniplates on the incidence of the remaining outcomes: wound dehiscence, infection, paresthesia, and nonunion/malunion. Subgroup analyses indicated that 3D miniplate caused a lower hardware failure than standard with the size of 8 or 10 holes (RR 0.23, 95% CI 0.08–0.66, *P* = 0.006). Besides, publication bias was not detected.

**Conclusion:**

The 3D miniplate is superior to the standard miniplate on the reduction of postoperative complication rates for the management of MFs. More holes in the 3D miniplate might contribute to a successful treatment.

## Background

Mandibular fractures (MFs) are the second, most-frequent facial injuries that account for 15.5–59% of all facial fractures [1]. The miniplate osteosynthesis that provides stable fixation contributing to bone alignment and healing was first introduced by Michelet in 1973 and further developed by Champy in 1975 [2]. It is considered as a standard surgical treatment of MFs [3]. However, debates on the stability of this single-miniplate fixation for the repair of the angle fractures remain and two plates in symphyseal or parasymphyseal region have been implied to counter increase the torsional forces [4]. A century ago, an aluminum-made quadrangular plate with bone screws at the lower border of the mandible was recommended by Lambotte and considered being superior to wiring osteosynthesis for the management of MFs [5]. However, due to the lower biocompatibility of the material and the preference of closed reduction, this method has not been widely applied. Thereafter, various plating approaches intraoral or extraoral have been reported to stable internal fixation [6], and the common treatment for MFs is the standard Champy miniplate fixation [7]. Recently, the 3-dimensional (3D) miniplate, which consists of several holes miniplates interconnected by vertical cross struts, has been introduced into the treatment of MFs [8]. Characterized by the geometrically stable configuration, 3D plating system allows the easy adaptation of plate to bone without distortion, which contributes to meeting the requirements of semirigid fixation with lesser complications [9].

Although a spectrum of studies has compared the efficiency of the 3D miniplate and the standard miniplate in the management of MFs [8–10], the optimum treatment was not defined due to different study designs, small sample sizes, and other factors. More recently, a review has been conducted by Al-Moraissi et al. to test whether there is a significant difference in the clinical outcomes between standard and 3D miniplate fixation in the management of mandibular angle fractures (MAFs) [11]. However, their meta-analysis only included 3 randomized controlled trials (RCTs) and 3 retrospective studies publishing in 2012–2013, and contained a relatively small sample size [11]. Besides, they also clarified the necessity of further investigation to reliably evaluate the postoperative complication rates between these two techniques without the isolation of MAF. Therefore, we retrieved the electronic databases up to August, 2015 and consequently included a set of 9 RCTs and 4 controlled clinical trials (CCTs), which were published in 2010–2015, in this meta-analysis to further compare the efficiency of these two miniplate fixations on postoperative complication rates in the management of MFs, attempting to provide a more reliable conclusion and determine the optimal strategy.

## Methods

As the paper did not involve any human or animal, the ethical approval was not required.

### Data source and search strategy

We retrieved the studies in several electronic databases including PubMed, Embase, Springer Link, and the Cochrane Library from their inception to August, 24th, 2015, with the key searching terms of “conventional” OR “champy” OR “champys” OR “standard” OR “linea oblique” AND “3-dimensional” OR “3D” OR “3-D” OR “strut” OR “grid”) AND “mandibular” OR “jaw.” A manual bibliographic search was also conducted to select additionally eligible studies. No language restriction was considered.

### Study selection

Two investigators independently reviewed the studies based on the following criteria and determined the eligibility of all the identified studies in the preliminary search. The inclusion criteria were as follows: (1) the participants in the studies were patients with MFs; (2) the 3D miniplate fixation was designed as experimental group, while the standard miniplate fixation was as control group; and (3) one of the following outcomes were included in the study: infection, malocclusion, hardware failure, nonunion/malunion, paresthesia, and wound dehiscence. On the contrary, studies were excluded if (1) data in the study were incomplete; (2) the measurement of bone mineral density in the study was inconsistent with the inclusion criteria; and (3) the study was a review, letter, comment, or case report.

### Data extraction and quality assessment

The required data for the eligible studies were independently abstracted by two investigators using a predefined form, which contained the following information: name of first author, year of publication, regions of the population, age and gender composition of patients, sample sizes, and study outcomes. The disagreement was resolved through a discussion involving a third investigator.

Quality assessment of the included studies was performed by the Cochrane Collaboration’s tools [12], which are used for assessing quality and risk of bias and involve seven items including random sequence generation, allocation concealment, blinding of participants and personnel, blinding of outcome assessment, incomplete outcome data, selective reporting, and other bias. This rigorous evaluation system makes the assessment more objective and comprehensive.

### Statistical analysis

Relative risk (RR) with its corresponding 95% confidence interval (CI) was selected as a measure of the pooled effect size of the treatment outcomes. Heterogeneity among studies was evaluated by using Cochran-based *Q* test and *I* ^2^ statistic [12]. If substantial heterogeneity was presented (*P* < 0.05, *I* ^2^ > 50%), the random effects model was selected to calculate the pooled effect size; otherwise, the fixed effects model was implemented when there lacked pronounced heterogeneity (*P* ≥ 0.05, *I* ^2^ ≤ 50%) [13]. Publication bias was measured by using a funnel plot. Subgroup analyses according to different fracture locations and different sites of 3D miniplate were considered. All the above statistical analysis was conducted using Review Manager 5.2 software (Cochrane Collaboration, http://ims.cochrane.org/revman).

## Results

### Literature search

**Fig. 1:**
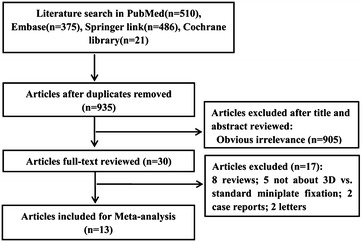
Flow chart of study selection

Based on the aforementioned search strategy, a total of 1392 studies were identified from preliminary search, including 510 from PubMed, 375 from Embase, 486 from Springer Link, and 21 from the Cochrane Library. After excluding the duplicate publications and removing the irrelevant articles via title browsing and abstract reading, the remaining 30 studies were put through full-text reading. Subsequently, another 17 articles were eliminated (8 reviewers, 2 case reports, 2 letters, and 5 studies that did not mention the curative effect comparison between 3D miniplate and standard miniplate). No additional articles were included using manual search strategy. Consequently, a set of 13 studies [8–10, 14–23] were included for this meta-analysis. The detailed procedure of study selection is presented in Fig. 1.

### Study characteristics

There were 9 RCTs [9, 10, 14–20] and 4 CCTs (3 retrospective studies [21–23] and 1 prospective study [8]) in this meta-analysis, consisting of 593 participants (339 in 3D miniplate group and 254 in standard miniplate group). The characteristics of the 13 included studies are summarized in Table 1. It indicated that they were published from 2010 to 2015, and the studies were conducted in the regions such as India (8 studies), American (3 studies), Germany (1 study), and Yemen (1 study). Moreover, the majority of the participants were males and there was not a significant difference between genders. In addition, the common most type of MFs was MAF, and others were symphysis, parasymphysis, condyle, ramus, and body. Major size of the 3D miniplate was 4 or 6 holes, and the else were 8 or 10 holes.

**Table 1:** Characteristics of studies included in the meta-analysis

Study	Year	Country	N (3D/Standard)	Design	Infection	Malocclusion	Hardware failure	Nonunion/malunion	Wound dehiscence	Paresthesia	Location (anterior, symphysis, parasymphysis)	The size of the steel plate
Al-Moraissi	2015	Yemen	20 (10/10)	RCT	1/0	NR	0/1	0/0	0/1	0/0	Mandibular angle fracture	8 hole
Agarwal	2014	India	80 (40/40)	RCT	2/0	NR	0/0	NR	NR	0/0	Symphysis, Parasymphysis	NA
Sehgal	2014	India	30 (15/15)	RCT	0/0	6/9	NR	0/0	NR	NR	Condyle, Symphysis, Parasymphysis, Body, Angle	4 hole
Sadhwani	2013	India	28 (14/14)	RCT	0/1	0/2	NR	0/0	NR	0/0	Symphysis, Parasymphysis, Angle	4 hole, 6 hole
Vineeth.	2013	India	20 (10/10)	RCT	0/2	2/5	0/1	NR	NR	2/1	Mandibular angle fracture	4 hole, 6 hole, 8 hole
Xue	2013	USA	11 (5/6)	RCT	0/0	NR	0/1	0/0	0/0	NR	Mandibular angle fracture	10 hole
Malhotra	2012	India	20 (10/10)	RCT	1/2	1/3	NR	0/0	NR	NR	Symphysis, Parasymphysis, body, Angle angle region indicated	4 hole
Singh	2012	India	50 (25/25)	RCT	2/3	NR	1/0	NR	NR	4/5	Symphysis, Parasymphysis, Angle	6 hole
Jain	2010	India	40 (20/20)	RCT	2/0	NR	NR	NR	0/0	NR	Symphysis, Parasymphysis, Body, Angle	4 hole, 6 hole, 8 hole
Barde	2014	India	40 (20/20)	PCS	2/3	1/4	NR	NR	0/3	1/1	Mandibular anterior fractures	4 hole
Moore	2013	USA	104 (72/32)	RCS	2/0	NR	2/6	3/1	1/0	NR	Mandibular angle fracture	8 hole
Guy, W. M.	2013	USA	90 (68/22)	RCS	3/2	2/2	1/1	3/0	4/2	1/1	Parasymphysis, Body, Angle, Ramus, Condyle, Coronoid process	NA
Hofer	2012	Germany	60 (30/30)	RCS	0/3	NR	0/2	0/0	0/3	NR	Mandibular angle fracture	6 hole

*M* male, *F* female, *RCT* randomized controlled trial, *PCS* prospective controlled study, *RCS* retrospective controlled study, *NR* not reported

### Risk of various bias and quality of studies

**Fig. 2:**
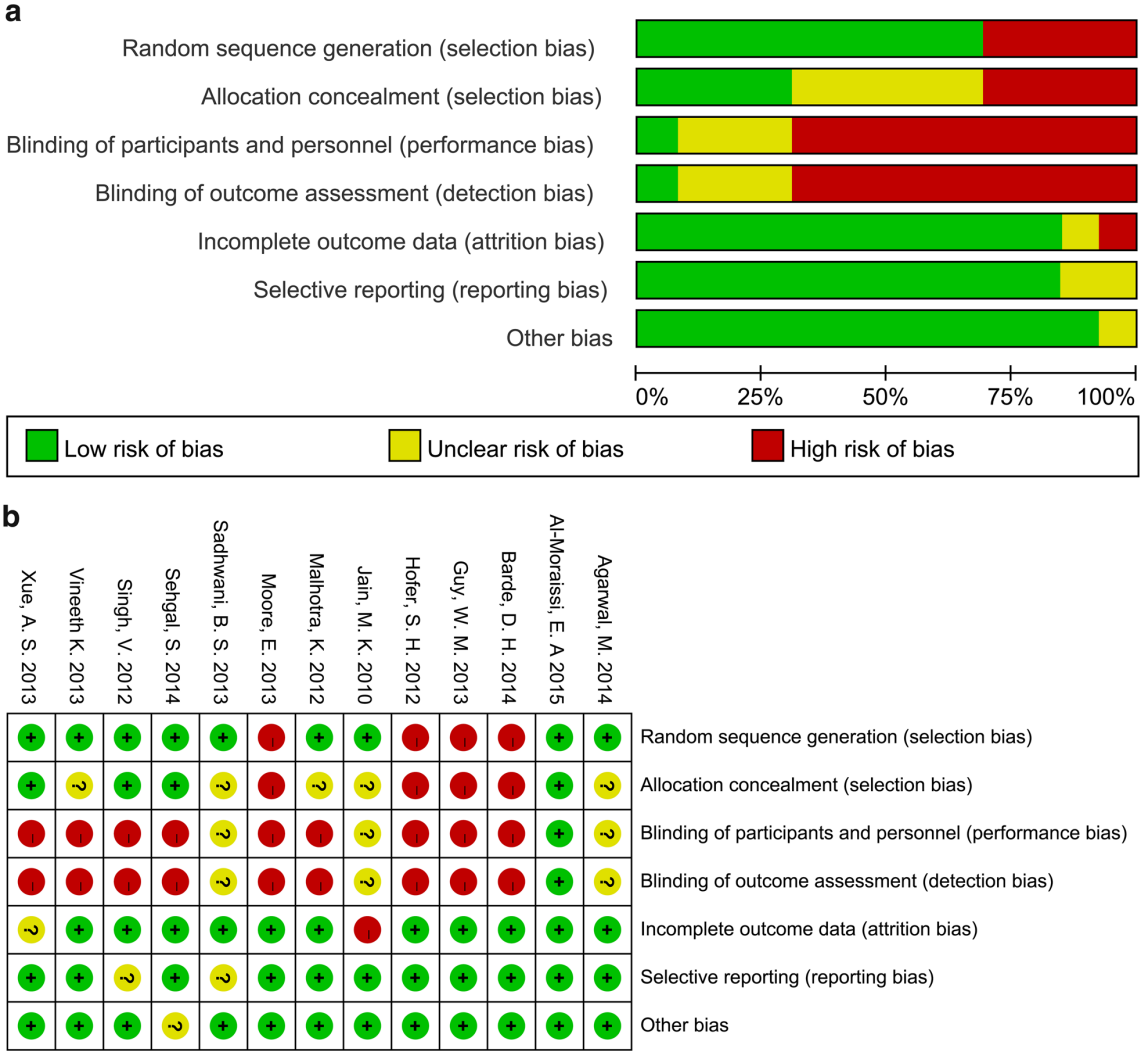
Assessment of the risk of bias for the included studies. **a** Methodological quality graph: authors’ judgment about each methodological quality item presented as percentages across all included studies; **b** methodological quality summary: authors’ judgment about each methodological quality item for each included study. +: low risk of bias; “?”: unclear risk of bias; “−”: high risk of bias

The quality assessment of the included studies is shown in Fig. 2. For most RCTs did not explicitly elucidate the randomized methods or mention the blinding and allocation concealment, the risk of selection bias, performance bias, and detection bias were relatively high. On the contrary, the risk of attrition bias, reporting bias, and other bias were relatively low. In summary, the overall bias risk was medium, and the quality of the 9 RCTs was considered as moderate. On the other hand, due to the high risk of performance bias and detection bias, quality of the 4 CCTs was deemed as low.

### Comparison of outcomes between standard miniplate and 3D miniplate in the management of MFs

**Fig. 3:**
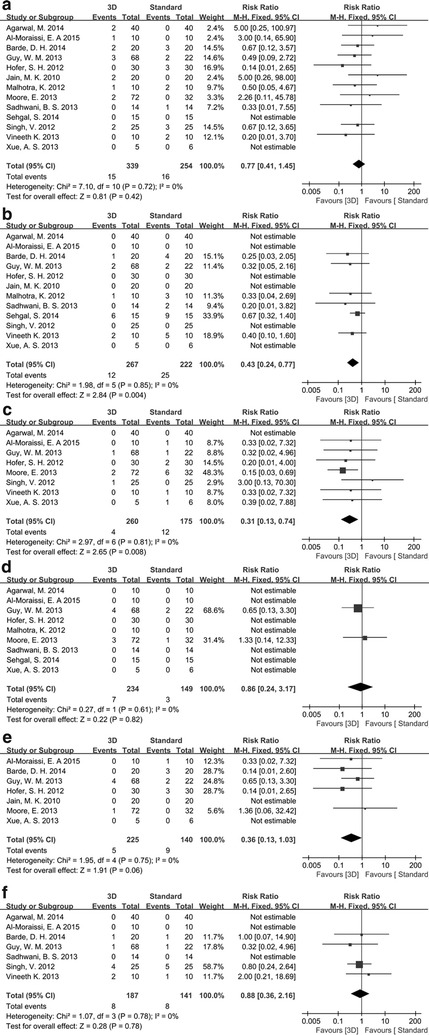
Forest plots of the effect comparisons between standard miniplate and 3-dimensional (3D) miniplate. **a** Infection; **b** malocclusion; **c** hardware failure; **d** nonunion/malunion; **e** wound dehiscence; **f** paresthesia. *Squares* represent the study-specific relative risk (RR) estimates, and the size of square reflects the study-specific weight. *Horizontal lines* represent 95% confidence interval (CI). The *diamond* represents the summary RRs with corresponding 95% CI

As a result, no significant heterogeneity among studies was presented (*I* ^2^ = 0%, *P* > 0.05) for evaluation of all the outcomes; thus, the fixed effects model was applied to calculate the pooled RRs (Fig. 3).

All the 13 studies reported the outcome of infection, and the overall RR for 3D miniplate vs standard miniplate was 0.77 (95% CI 0.41–1.45, *P* = 0.42, Fig. 3a), suggesting a comparable effect between the two miniplates.

The malocclusions were examined in 12 studies, and 3D miniplate achieved a significantly lower incidence of malocclusion (RR 0.43, 95% CI 0.24–0.77, *P* = 0.004, Fig. 3b) than the standard miniplate.

Additionally, 3D miniplate pronouncedly decreased the effect of hardware failure from the pooled results of 7 studies (RR 0.31, 95% CI 0.13–0.74, *P* = 0.008, Fig. 3c).

Nine studies reported the outcomes of nonunion/malunion and the combined result indicated that 3D miniplate achieved a lower incidence of this outcome (RR 0.86, 95% CI 0.24–3.17, *P* = 0.82, Fig. 3d), however, without significant difference.

The wound dehiscence was involved in 6 studies. Likewise, incidence of this outcome was lower with the treatment of 3D miniplate than with the standard miniplate (RR 0.36, 95% CI 0.13–1.03, *P* = 0.06, Fig. 3e), however, without statistical significance.

Seven studies determined the outcome of paresthesia, and no pronounced differences were observed between the two miniplates (RR 0.88, 95% CI 0.36–2.16, *P* = 0.78, Fig. 3f).

Notably, for the two outcomes of infection and malocclusion, we combined the results of all the RCTs and found that the newly pooled results were consistent with the overall analysis (infection: RR 1.00, 95% CI 0.44–2.28, *P* = 1.00; malocclusion: RR 0.49, 95% CI 0.26–0.91, *P* = 0.02).

### Subgroup analysis results

When stratified by different fracture regions, MAF and others, the results of most outcomes were the same with the overall results, except malocclusion (Table 2). Unexpected, in MAF subgroup, the reduced effect of 3D miniplate on malocclusion was not significant, compared with the standard miniplate (RR 0.40, 95% CI 0.10–1.60, *P* = 0.20), whereas in other fracture regions 3D also had a reduced effect (RR 0.44, 95% CI 0.23–0.83, *P* = 0.01). Results in terms of wound dehiscence were similar to the overall result, either in the MAF subgroup or the other regions subgroup. With regard to the hardware failure, in MAF subgroup, 3D miniplate achieved a significantly lower outcome than the standard (RR 0.22, 95% CI 0.07–0.63, *P* = 0.005), while in the subgroup of other regions, there were no significant differences.

**Table 2:** Subgroup analysis stratified by different locations and different steel plate sites

Indicator	Group	Sample size		Test of association			Model	Test of heterogeneity^a,b^		
		Cases	Control	RR (95% CI)	*Z*	*P* value		Chi^2^	*P* value	*I* ^2^ (%)
Fracture regions										
Infection	Angle	127	88	0.56 [0.17, 1.89]	0.93	0.35	Fixed	3.28	0.35	8
	Other	212	166	0.88 [0.42, 1.84]	0.33	0.74	Fixed	3.89	0.69	0
Malocclusion	Angle	55	56	0.40 [0.10, 1.60]	1.30	0.20	–	–	–	–
	Other	212	166	0.44 [0.23, 0.83]	2.53	0.01	Fixed	1.91	0.75	0
Hardware failure	Angle	127	88	0.22 [0.07, 0.63]	2.82	0.005	Fixed	0.53	0.97	0
	Other	133	87	0.99 [0.16, 5.99]	0.01	0.99	Fixed	1.12	0.29	11
Nonunion/malunion	Angle	117	78	1.33 [0.14, 12.33]	0.25	0.80	–	–	–	–
	Other	117	71	0.65 [0.13, 3.30]	0.52	0.60	–	–	–	–
Wound dehiscence	Other	108	62	0.38 [0.09, 1.49]	1.39	0.16	Fixed	0.85	0.36	0
	Other	108	62	0.38 [0.09, 1.49]	1.39	0.16	Fixed	0.85	0.36	0
Paresthesia	Angle	20	20	2.00 [0.21, 18.69]	0.61	0.54	–	–	–	–
	Other	167	121	0.73 [0.27, 1.99]	0.61	0.54	Fixed	0.42	0.81	0
The size of the steel plate										
Infection	4 or 6 hole	114	114	0.46 [0.18, 1.16]	1.64	0.10	Fixed	1.03	0.91	0
	Other	225	140	1.30 [0.53, 3.21]	0.57	0.57	Fixed	4.81	0.44	0
Malocclusion	4 or 6 hole	114	114	0.46 [0.23, 0.90]	2.26	0.02	Fixed	1.68	0.64	0
	Other	135	108	0.37 [0.12, 1.14]	1.74	0.08	Fixed	0.03	0.86	0
Hardware failure	4 or 6 hole	55	55	0.67 [0.11, 3.89]	0.45	0.65	Fixed	1.49	0.22	33
	Other	205	120	0.23 [0.08, 0.66]	2.75	0.006	Fixed	0.60	0.96	0
Nonunion/malunion	4 or 6 hole	39	39	–	–	–	–	–	–	–
	Other	195	110	0.86 [0.24, 3.17]	0.22	0.82	Fixed	0.27	0.61	0
Wound dehiscence	4 or 6 hole	50	50	0.14 [0.02, 1.12]	1.85	0.06	Fixed	0.00	1.00	0
	Other	175	90	0.65 [0.18, 2.36]	0.65	0.51	Fixed	0.39	0.82	0
Paresthesia	4 or 6 hole	59	59	0.83 [0.28, 2.48]	0.33	0.74	Fixed	0.02	0.88	0
	Other	128	82	0.99 [0.21, 4.79]	0.01	0.99	Fixed	1.03	0.31	2

*RCT* randomized controlled trial, *PCS* prospective controlled study, *RCS* retrospective controlled study

^a^Random effects model was used when the *P* value for heterogeneity test <0.05, otherwise the fixed effects model was used

^b^ *P* value <0.05 is considered statistically significant for Q statistics

When stratified by different 3D miniplate sizes, 4 or 6 holes and others, results on almost all the outcomes were not significantly different except malocclusion and hardware failure. In 4 or 6 hole subgroup, 3D had a significantly reduced malocclusion than the standard miniplate (RR 0.46, 95% CI 0.23–0.90, *P* = 0.02). In other size subgroup (8 or 10 holes), 3D significantly decreased the hardware failure compared with the standard miniplate (RR 0.23, 95% CI 0.08–0.66, *P* = 0.006).

### Publication bias

**Fig. 4:**
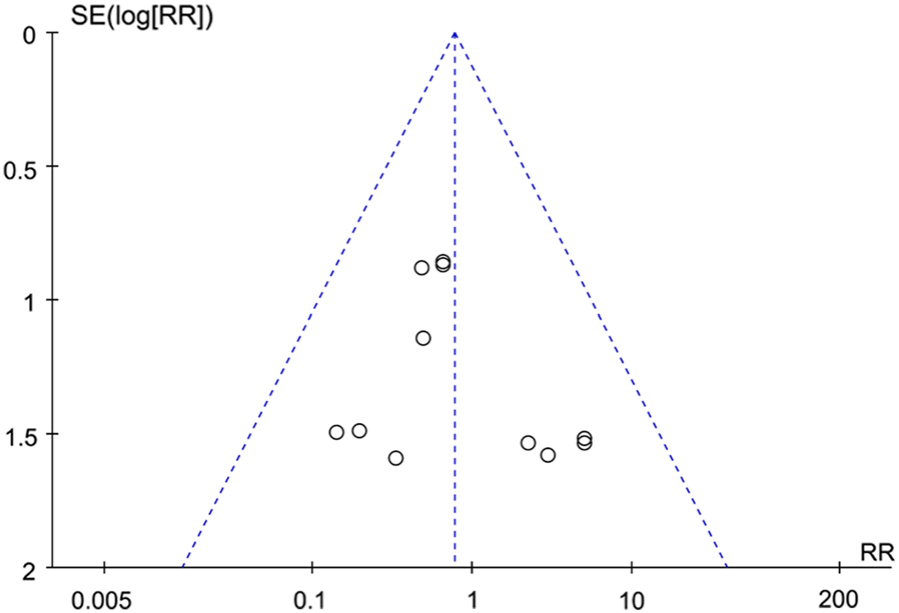
Funnel plot for publication bias test of the comparison between standard miniplate and 3D miniplate in infection

The publication bias for the outcome of infection, which was involved in 13 studies, was estimated. As revealed in the funnel plot (Fig. 4), the obvious asymmetry was not observed, indicating the absence of publication bias in this meta-analysis.

## Discussion

This meta-analysis included a set of 13 studies to evaluate the efficiency of the standard miniplate and 3D miniplate on the treatment of MFs. As a result, we found that 3D miniplate significantly decreased the incidence of malocclusion and hardware failure, compared with the standard miniplate. However, there did not detect any pronounced difference between these two treatments with regard to the outcomes of infection, nonunion, and paresthesia. Although 3D miniplate attained a dramatic decrease on wound dehiscence compared with the standard, no significant differences were observed (*P* = 0.06). Moreover, the pooled results of all the RCTs also exhibited a lower malocclusion incidence than the standard treatment. Subgroup analysis indicated that when stratified by different fracture regions, results on almost all the outcomes were the same as the overall results, except the malocclusion; when stratified by different 3D miniplate sizes, in other size subgroup (8 holes or 10 holes), 3D significantly decreased the hardware failure compared with the standard.

For the management of MFs, the failure to achieve a stable condition in the right anatomical position that enables the undisturbed healing could result in malocclusion, infection or nonunion [14]. The 3D miniplate has the advantage of the simultaneous stabilization of the tension and compression zones [11], which might contribute to the lower incidence of postoperative complications and the good clinical results [24].

The outcome of malocclusion is evaluated by several studies comparing different miniplates for MFs. However, most of the studies find that there is not any pronounced difference between the two miniplates for MAFs [11, 25, 26], or that the incidence of malocclusion is not detected with both of the two miniplates [20]. While in the present meta-analysis, all the studies concerning the malocclusion [8, 14, 17–19, 22] showed a relatively lower incidence with the 3D miniplate than with the standard miniplate. Moreover, the combined results indicated that 3D miniplate significantly decreased the incidence of malocclusion (RR 0.43, 95% CI 0.24–0.77, *P* = 0.004), compared with the standard miniplate. Notably, the combined results of the 9 RCTs indicated that 3D miniplate pronouncedly reduced the incidence of malocclusion, comparing with the standard miniplate. These collectively favored the advantage of 3D miniplate in the management of MFs. Unexpectedly, in the MAF subgroup, although 3D had a reduced effect on malocclusion compared with the standard, the difference was not significant. The plausible reason is that MAF is accounting for 30% of all the MFs, and any additional fracture may cause the instability at the fracture site, which may consequently impair the bone healing and be prone to malocclusion [11, 25]. In addition, in our study, the above studies that concerned malocclusion in our meta-analysis were not MAF, while most studies in the MAF subgroup did not evaluate the malocclusion outcome, which might reasonably explained the undesirable result without significance.

It has been indicated that the infection rate of patients with angle fractures using 3D miniplate was as low as 9% [27] or 8.2% [28]. Nevertheless, different results of the comparison between 3D miniplate and the standard miniplate on the incidence of infection were presented. Several studies observed that the infection rate of 3D miniplate was relatively lower than the standard [9, 23], while others indicated a comparable efficiency between these two techniques [10, 16], which might be due to the excessive implant material [16]. Notably, the pooled results in this meta-analysis exhibited a 23% induction of the incidence of infection rate using 3D miniplate, compared to the standard method, though without significant difference.

Hardware failure is a significant complication of MFs [6]. Although 3D miniplate was testified stable for the treatment of simple MAFs with low complication rates [29], its hardware-related advantages over the conventional miniplates were only emphasized by a small handful of studies [30, 31]. Due to the limited samples, hardware failure is not detected in several cases, therefore, several studies discover that there are no significant differences between 3D and the standard miniplates for the management of MFs [20, 26]. However, a meta-analysis favors the use of 3D miniplate for it could pronouncedly decrease the incidence of hardware failure, when compared to the standard miniplate for the treatment of MAFs (RR 0.18, 95% CI 0.05–0.6, *P* = 0.005) [11]. In accordance with this study, we found that the incidence of hardware failure, based on the overall result or the MAF subgroup result, was markedly lower using the 3D miniplate than the standard. Notably, when stratified by different 3D miniplate sizes, it was found in other size (8 holes or 10 holes) subgroup, the hardware failure was significantly decreased applying 3D than standard. These collectively supported the superiority of 3D miniplate. Moreover, based on the subgroup analysis by size, it might be inferred that large size of the 3D miniplate with more holes could contribute to the successful treatment.

According to the Champy technique, the standard miniplate needs to be placed on the external oblique line, which leads to the proximity to incision, whereas the 3D miniplate could easily avoid this situation as it is covered by the masseter along the buccal cortex, well away from the incision [15, 21], and this might be the reason for differences between the two miniplates in the incidence of wound dehiscence. The pooled results integrating 6 studies in this meta-analysis indicated a dramatic reduction (64%) in the incidence of wound dehiscence using 3D miniplate than the standard, however, without significant difference (*P* = 0.07), which might be due to the small sample size.

The displacement of the segments is the major cause that leads to sensory abnormalities. Thus, paresthesia might be detected in all the miniplates after surgery [11]. For most studies that did not detect significant differences between these two miniplates, the pooled results based on 7 studies in the present meta-analysis revealed a minor decrease in the incidence of paresthesia, without significant difference. Moreover, in either of the subgroups stratified by different fracture regions, the results were the same.

Although the study favored the 3D miniplate in the management of MFs and there lacked pronounced heterogeneity, several limitations should be discussed. Although more RCTs and CCTs were included in the present meta-analysis, the sample sizes remained small. Besides, the quality of the CCTs was considered as low for most of them did not mention the blind design. Moreover, several outcomes such as nonunion/malunion, malocclusion, and wound dehiscence need to be further assessed because the incidences of these factors were not detected in most studies. Therefore, more high-quality RCTs with larger sample sizes were required to provide more precise assessment. In addition, 3D miniplate is much more complicated, leads to larger incisions, and has a much higher cost than the standard miniplate. There are a multitude of fractures types among MFs (such as condylar neck fractures, angle fractures, or parasymphyseal fractures); thus, the fracture type being more suitable with 3D miniplate should be deeply explored.

In conclusion, our meta-analysis suggests that 3D miniplate is superior to the standard miniplate on the reduction of postoperative complication rates in the management of MFs. The size of 3D miniplate with more holes (8 or 10) might contribute to the successful treatment. However, more high-quality RCTs are warranted to confirm these findings, and the MF subtype being more suitable with 3D miniplate should be deeply explored.

## Abbreviations

3D: 3-dimensional
MFs: mandibular fractures
RR: relative risk
CI: confidence interval
MMF: maxillomandibular fixation
ORIF: open reduction and internal fixation
RCT: randomized control trial
CCT: clinical cohort trial

## References

[1] Andreas ZJ, Benoit S, Olivier L, Nikola S, Hanna T, Tateyuki I (2011). Incidence, aetiology and pattern of mandibular fractures in central Switzerland. Swiss Med Wkly.

[2] Champy M, Wilk A, Schnebelen J (1975). Die Behandlung der Mandibular-frakturen mittels Osteosynthese ohne intermaxillare Ruhigstellung nach der Technik von FX Michelet. Zahn Mund Kieferheilkd Zentralbl..

[3] Singh V, Kumar I, Bhagol A (2011). Comparative evaluation of 2.0-mm locking plate system vs 2.0-mm nonlocking plate system for mandibular fracture: a prospective randomized study. Int J Oral Maxillofac Surg.

[4] Choi B, Yoo J, Kim K, Kang H (1995). Stability testing of a two miniplate fixation technique for mandibular angle fractures. An in vitro study. J Cranio Maxillofac Surg.

[5] Lambotte A. Chirurgie opératoire des fractures. Masson. 1913;1:556.

[6] Zix J, Lieger O, Iizuka T (2007). Use of straight and curved 3-dimensional titanium miniplates for fracture fixation at the mandibular angle. J Oral Maxillofac Surg.

[7] Gear AJ, Apasova E, Schmitz JP, Schubert W (2005). Treatment modalities for mandibular angle fractures. J Oral Maxillofac Surg.

[8] Barde DH, Mudhol A, Ali FM, Madan R, Kar S, Ustaad F (2014). Efficacy of 3-dimensional plates over Champys miniplates in mandibular anterior fractures. J Int Oral Health.

[9] Singh V, Puri P, Arya S, Malik S, Bhagol A (2012). Conventional versus 3-dimensional miniplate in management of mandibular fracture a prospective randomized study. Otolaryngol Head Neck Surg.

[10] Agarwal M, Meena B, Gupta D, Tiwari AD, Jakhar SK (2014). A prospective randomized clinical trial comparing 3D and standard miniplates in treatment of mandibular symphysis and parasymphysis fractures. J Maxillofac Oral Surg.

[11] Al-Moraissi E, El-Sharkawy T, El-Ghareeb T, Chrcanovic B (2014). Three-dimensional versus standard miniplate fixation in the management of mandibular angle fractures: a systematic review and meta-analysis. Int J Oral Maxillofac Surg.

[12] Higgins JP, Green S, editors. Cochrane handbook for systematic reviews of interventions. Chichester: Wiley-Blackwell; 2008.

[13] Liu Y-J, Zhan J, Liu X-L, Wang Y, Ji J, He Q-Q (2014). Dietary flavonoids intake and risk of type 2 diabetes: a meta-analysis of prospective cohort studies. Clin Nutr.

[14] Vineeth K, Lalitha R, Prasad K, Ranganath K, Shwetha V, Singh J (2013). “A comparative evaluation between single noncompression titanium miniplate and three dimensional titanium miniplate in treatment of mandibular angle fracture”—a randomized prospective study. J Cranio Maxillofac Surg.

[15] Xue AS, Koshy JC, Wolfswinkel EM, Weathers WM, Marsack KP, Hollier LH (2013). A prospective study of strut versus miniplate for fractures of mandibular angle. Craniomaxillofac Trauma Reconstr.

[16] Jain MK, Manjunath K, Bhagwan B, Shah DK (2010). Comparison of 3-dimensional and standard miniplate fixation in the management of mandibular fractures. J Oral Maxillofac Surg.

[17] Sadhwani BS, Anchlia S (2013). Conventional 2.0 mm miniplates versus 3-D plates in mandibular fractures. Ann Maxillofac Surg.

[18] Sehgal S, Ramanujam L, Prasad K, Krishnappa R (2014). Three-dimensional v/s standard titanium miniplate fixation in the management of mandibular fractures—a randomized clinical study. J Cranio Maxillofac Surg.

[19] Malhotra K, Sharma A, Giraddi G, Shahi AK (2012). Versatility of titanium 3D plate in comparison with conventional titanium miniplate fixation for the management of mandibular fracture. J Maxillofac Oral Surg.

[20] Al-Moraissi E, Mounair R, El-Sharkawy T, El-Ghareeb T (2015). Comparison between three-dimensional and standard miniplates in the management of mandibular angle fractures: a prospective, randomized, double-blind, controlled clinical study. Int J Oral Maxillofac Surg.

[21] Moore E, Bayrak S, Moody M, Key JM, Vural E (2013). Hardware removal rates for mandibular angle fractures: comparing the 8-hole strut and Champy plates. J Craniofac Surg.

[22] Guy WM, Mohyuddin N, Burchhardt D, Olson KL, Eicher SA, Brissett AE (2013). Repairing angle of the mandible fractures with a strut plate. JAMA Otolaryngol Head Neck Surg.

[23] Höfer SH, Ha L, Ballon A, Sader R, Landes C (2012). Treatment of mandibular angle fractures–Linea obliqua plate versus grid plate. J Cranio Maxillofac Surg.

[24] Chrcanovic BR (2014). Fixation of mandibular angle fractures: clinical studies. Oral Maxillofac Surg.

[25] Al-Moraissi EA, Ellis E (2014). What method for management of unilateral mandibular angle fractures has the lowest rate of postoperative complications? A systematic review and meta-analysis. J Oral Maxillofac Surg.

[26] Al-Moraissi EA, Ellis E (2014). Surgical management of anterior mandibular fractures: a systematic review and meta-analysis. J Oral Maxillofac Surg.

[27] Feledy J, Caterson EJ, Steger S, Stal S, Hollier L (2004). Treatment of mandibular angle fractures with a matrix miniplate: a preliminary report. Plast Reconstr Surg.

[28] Bui P, Demian N, Beetar P (2009). Infection rate in mandibular angle fractures treated with a 2.0-mm 8-hole curved strut plate. J Oral Maxillofac Surg.

[29] Hochuli-Vieira E, Ha TKL, Pereira-Filho VA, Landes CA (2011). Use of rectangular grid miniplates for fracture fixation at the mandibular angle. J Oral Maxillofac Surg.

[30] Parmar S, Menat S, Raghani M, Kapadia T (2007). Three dimensional miniplate rigid fixation in fracture mandible. J Maxillofac Oral Surg..

[31] Guimond C, Johnson JV, Marchena JM (2005). Fixation of mandibular angle fractures with a 2.0-mm 3-dimensional curved angle strut plate. J Oral Maxillofac Surg.

